# Study on the Evaluation and Assessment of Ecosystem Service Spatial Differentiation at Different Scales in Mountainous Areas around the Beijing–Tianjin–Hebei Region, China

**DOI:** 10.3390/ijerph20021639

**Published:** 2023-01-16

**Authors:** Hui Yang, Jiansheng Cao, Xianglong Hou

**Affiliations:** 1 Key Laboratory of Agricultural Water Resources, Hebei Key Laboratory of Agricultural Water-Saving, Center for Agricultural Resources Research, Institute of Genetics and Developmental Biology, Chinese Academy of Sciences, Shijiazhuang 050001, China; 2 Institute of Geographical Sciences, Hebei Academy of Sciences, Shijiazhuang 050011, China; 3 Hebei Technology Innovation Center for Geographic Information Application, Shijiazhuang 050011, China

**Keywords:** ecosystem service, human wellbeing, land use land cover, Taihang Mountain, Yanshan Mountain, Bashang region

## Abstract

Ecosystem services are closely related to human wellbeing—which refers to the basic material conditions required to maintain high standards of quality of life, of health, and of safety and security, as well as good social relationships, and freedom of choice and action—and have a high potential value. The adequate valuation of ecosystem service values (ESV) is now an urgent need for the implementation of policy measures such as ecosystem asset management, ecological compensation and paid use of ecosystem services. Based on the method of ecosystem value equivalent factor per unit area, in this paper we investigated the variability of total ESV spatial distribution and regional differences in the value of 11 ecosystem service types provided by ecosystems in the mountainous areas of the Beijing–Tianjin–Hebei (BTH) region at different spatial scales and discussed the relationship between ESV and land use land cover (LULC). The results show that the total ESVs in 2015 and 2020 in the mountainous areas of BTH region were 5671 × 10^8^ CNY and 6381 × 10^8^ CNY, respectively. The change trend of each ecosystem service value from 2015 to 2020 was dominated by an increase of water supply service, and the maintenance of soil and nutrient cycle services in the mountainous area of BTH and the Yanshan Mountain (YSM). The change patterns of the value of ecosystem service categories in the Taihang Moutain (THM) and Bashang region (BSR) are dominated by an increase in the value of maintained soil and water supply services and a decrease in the value of regulating services. The calculation of ESV can be made more accurate by considering climate and vegetation conditions at the region, ecosystem, county or township scales rather than at grid scale, as, for calculating the interannual variation of total ESV, the spatial scale variability is large. The assessment of ESVs is important from the point of view of planning the use of the environment, of spatial planning, and of nature conservation. The ecosystem service of woodland and water bodies are more prominent compared with other land-use types and construction land and bare land provide the lowest value of service. There is some similarity between the value changes of ecosystem service categories and the distribution of LULC types. This study strengthens the understanding of the spatial scales of different ESV in mountain areas, which is beneficial to ecosystem management policies.

## 1. Introduction

Research on the relationship between ecosystem services (ES) and human wellbeing is a key component of the Global Land Project (GLP) and the Future Earth Program (FEP) and is an important research direction for national and regional sustainable human development [1]. The concept of human wellbeing is abstract and has many interpretations [2]. In a broad sense, human wellbeing is divided into objective wellbeing and subjective wellbeing. Objective wellbeing consists of the objective material and social attributes of peoples’ living environments, such as material resources, housing, employment and income, education and health, etc. [3]. In contrast, subjective human wellbeing is a measure of one’s feelings, thoughts, and satisfaction with life circumstances, and is measured by psychological responses such as life satisfaction, freedom, social relationships, and personal safety [4]. ES is a bridge between natural systems and human wellbeing, and ecosystems continue to provide goods and services to people through ecological functions that meet human wellbeing. ES refers to the benefits that natural ecosystems directly or indirectly provide to people [5,6]. Furthermore, these services are closely associated with human wellbeing, health, livelihood, and survival [7,8]. ESs are generally subdivided into the categories of provisioning (e.g., food or energy outputs), regulating (biophysical processes providing regulatory benefits such as climate regulation, water regulation) and cultural services (e.g., recreational, aesthetic, spiritual benefits), which are all highly relevant to human wellbeing, as well as supporting services (referring to processes that are vital to the provision of other ecosystem services), which are fundamental to the maintenance of the above three types [9]. ESs are attributed to a unique socio-ecological perspective, which has replaced GDP as a new standard for assessing regional development [8]. In the past fifty years, global biodiversity has suffered serious damage, with a third of the world’s species at risk of extinction, while 60 percent of ecosystem services have been degraded, 10–20 percent of land has been degraded and only eight percent of land has access to adequate water. In order to meet the need for food, wood, fuel, etc., human beings have accelerated the alteration of ecosystems, resulting in the degradation of some ecosystems and directly threatening regional ecological security [10]. ES is a framework by which to integrate the relationship between ecosystems and people [11]. Therefore, for the purposes of policy and planning, the evaluation and valuation of ES is important to help decision-makers to allocate resources [12] and design policies related to ES [13]. The assessment of ES is also an important prerequisite and basis for the protection of the ecological environment and of biodiversity, as well as for ecological function zoning, environmental economic accounting, for the realization of an ecological civilization and for the response to various environmental problems worldwide [14,15,16,17].

Land use and land cover (LULC) is a direct manifestation of the transformation of nature by anthropogenic activities, as an expression of the close intersection between human society and natural ecological environments [18]. According to Lawler et al. [19] and Park et al. [20], conversion of the natural ecosystem into croplands, plantations, and urban areas increases food production, fiber, timber, and housing but has also caused a decline in other ecosystem services. Land-use and land-cover changes (LULCC) can directly affect the structure and function of an ecosystem [21,22] and play a decisive role in maintaining regional ecological balance. Changes in the status, characteristics, and functions of ecosystems inevitably affect the supply of ecosystem services and cause changes in ecosystem service values (ESVs) [23]. Combining LULCC and ESV data helps to identify the most vulnerable ecosystems and, to hence provide an entry point for land management [24]. Therefore, recognizing and estimating the effects of LULCC on global, regional, and local ESVs is a practical approach for the evaluation of costs and benefits of sustainable land management decisions [25,26,27]. It also aids in the advance of a land-use planning framework that is compatible with the long-term sustainability of land resources [28,29].

Mountain ecosystems provide a variety of important ESs to humanity, both to people living in the mountains and to people living away from the mountains [30,31]. They support the services of climate regulation, pollination, pest and weed control, retention, purification and nutrient cycling [32,33]. For example, the regulation ES provided by Kenyan montane forests, as an important production factor of agriculture, forestry, water, etc. [34,35], contributed approximately 33–39% of the local gross domestic product (GDP) between 2000 and 2010. Promoting the coordinated development of the Beijing–Tianjin–Hebei (BTH) region in China is an important national strategy; However, the regional ecological environment is the primary issue limiting the implementation of this strategy [36]. Mountainous areas around the BTH region are the main ecological security barrier and water conservation areas in northern China and comprise the only natural ecosystem that can be relied upon to achieve the goal of carbon neutrality in the BTH region. Mountainous areas around BTH region play an irreplaceable and important role in maintaining the regional ecological environment, guaranteeing water and food security, and is a key area in supporting the high-quality development of the BTH mega-city cluster. Existing studies indicate the importance of ESs in terms of net productivity (NPP), soil conservation (SC), habitat quality (HQ) and water yield (WY) in the mountainous areas around the BTH region [31]. However, most relevant studies have focused on only one aspect of the ecosystem’s characteristics and have discussed its impact factors separately [37], meaning that the assessment results are inconsistent [35]. In addition to the way that different assessment methods incur different results, vegetation also has a key role in the ecological processes and makes important contributions to the value of the relevant ESs. This is important because the mountainous areas around the BTH region have a strong heterogeneity of vegetation distribution and obvious changes in vegetation cover gradient [38].

Based on the global equivalent factor table proposed by Costanza et al. [5], a large number of studies on ecosystem service values (ESVs) have emerged [39,40]. ESVs have been assessed at different scales, including regions [41,42,43], basins [44,45,46], coastal areas [47,48], island [49], and cities [50,51], and for different ecosystem types such as forest [52,53], grassland [54], plateau [55], lake [56,57], wetland [58,59], farmland [60,61], and ocean [62]. Although a large amount of research work has been conducted on the valuation methods of ESVs [5,63,64], a unified valuation system has not yet been formed [65,66,67], and the differences in methods have led to large differences in research results, thus limiting the objective perception of ES functions and their values. At present, the accounting of ESVs can be broadly divided into two categories, namely, methods based on the price-per-unit service function (the functional value method) [68] and methods based on the equivalent factor per unit area value (the equivalent factor method) [63]. Compared with the functional value method, the equivalent factor method is more intuitive and easier. It is particularly suitable for the assessment of ecosystem service values at regional and global scales because it is easy to use and requires less data [69,70].

ESs exhibit complex interconnections and strong scale characteristics [30,71], accounting for natural capital [72] and gross ecosystem product [73], evaluate specific policies [74], plan for land use [75], and develop ES payment schemes [76]. As the basis of all ecological studies, scale has always been the focus and challenge of ecosystem services research [77,78]. The scale-linked nature of ES allows the same set of ES trade-off/synergistic relationships to vary across scales [35].

Thus, in this paper, we estimated the ESVs in mountainous areas of the Beijing–Tianjin–Hebei region in China in 2015 and 2020 at five types of spatial scales, including grid scale, township scale, county scale, regional scale (Yanshan Mountain region, Bashang region and Taihang Mountain region), and ecosystem scale. The objective of this paper is to evaluate and valuate ES at different spatial scales, to discuss the variability in ES across different spatial scales and to clarify the relationship between ESVs and LULC. The results can provide an important scientific and technological reference for research on eco-environmental protection, on environmental management decisions, on building greenfield cities and on supporting the sustainable development of the Beijing–Tianjin–Hebei region in China, as well as on the rationality of the selection of research and assessment methods related to ESVs in different regions and at different scales.

## 2. Materials and Methods

### 2.1. Study Area

**Figure 1:**
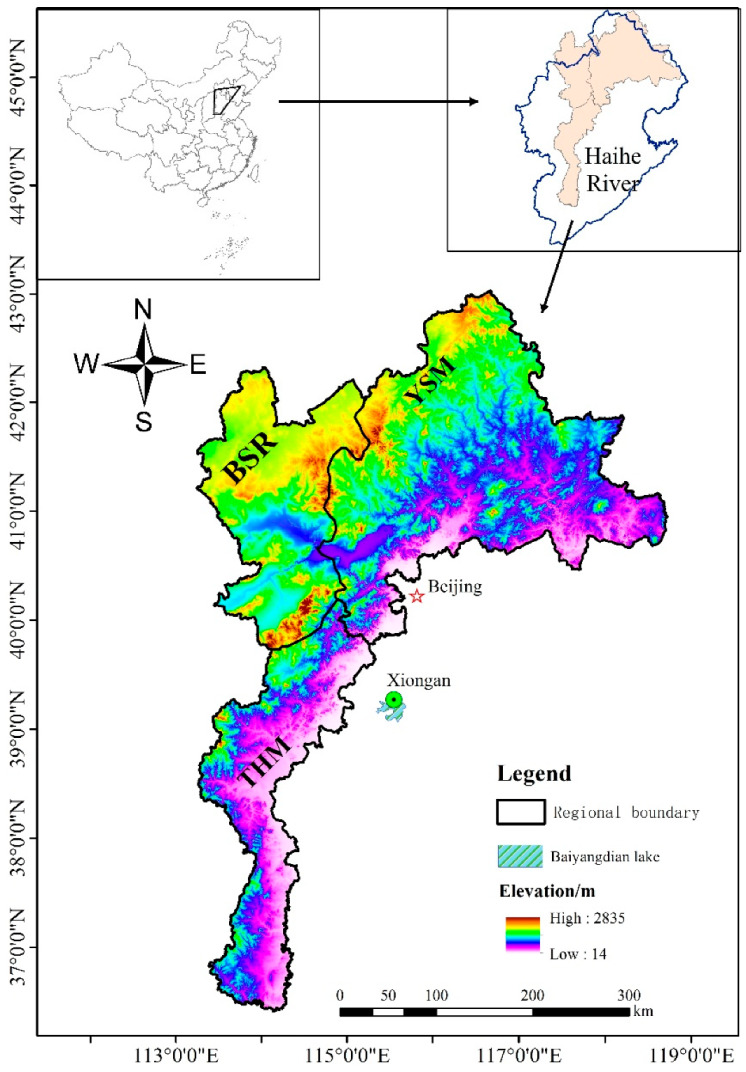
Location of study area (THM—Taihang Mountain, BSR—Bashang region, YSM—Yanshan Mountain).

The mountainous areas of BTH includes mainly Taihang Mountain (THM), Yanshan Mountain (YSM) and Bashang region (BSR), and form the basis of drinking water resources, ecological protection and green food supply of Beijing, Tianjin and Hebei. The total area of the mountainous area in BTH is 121,869 km^2^, of which the THM is 29,361 km^2^, accounting for 24% of the total area; the BSR is 29,610 km^2^, accounting for 24.6%; and the YSM is 62,899 km^2^, accounting for 51.4%. It is located at 113°30′0″–119°34′40″ E, 36°14′0″–42°38′10″ N, to the west of Beijing, Tianjin and Shijiazhuang, the capital of Hebei province. The upstreams of Xiongan New Area and Baiyangdian lake, and the Haihe River basin all flow through the area (Figure 1). It is a hilly area in the transition zone from plain to plateau and in the warm-temperate continental monsoon climate zone, which is hot and rainy in summer and cold and dry in winter. Additionally, the region has an average annual temperature of about 11 °C and average annual precipitation of about 500 mm, water surface evaporation is 1200 mm. The typical vegetation type is mainly 20-year-old secondary forest and scrub. The major soil type is cinnamon soil and the overlying soil layer is thin, with a thickness of 20–50 cm and mainly constituted of gravel. The thickness of the underlying rock layer, which is mainly a weathered layer of gneiss that is full of fissures, is 0.5–10 m.

### 2.2. Data and Method

The data of LULC in 2015 and 2020, with a resolution of 1 km, were downloaded from the Resources and Environmental Sciences Data Center (RESDC), Chinese Academy of Sciences (http://www.resdc.cn). The data of township and county boundary were also downloaded from http://www.resdc.cn. 

The data on terrestrial ESVs were derived from the Spatial Distribution of Terrestrial Ecosystem Service Values in China dataset [79], which is based on the national remote sensing classification of terrestrial ecosystem types. The ecosystem types include 15 secondary categories, namely dryland, farmland, coniferous forest, mixed coniferous forest, broadleaf forest, shrubland, grassland, scrub, meadow, wetland, desert, bare land, water body, glacial snow, and artificial surface (including construction land, industrial and mining land) and six primary categories, namely farmland, woodland, grassland, wetland, bare land and water body. Based on the spatial distribution of the net primary productivity of vegetation (NPP), precipitation, and soil conservation, the value of each service equivalent factor of the ecosystem was adjusted by referring to the ecological service equivalent factor method of Xie et al. [68]. Additionally, the values of food production, raw material production, water supply, gas regulation, climate regulation, environmental purification, hydrological regulation, soil maintenance, nutrient cycle maintenance, biodiversity maintenance, and landscape aesthetic were valued for a total of 11 ecological services. The data are presented in units of 10^4^ Chinese Yuan (CNY)/km^2^ and the spatial resolution is 1 km.

According to Xie et al. [68], first, the amount of economic value of the standard ecosystem ESV equivalent factor needs to be determined. That is, the economic value of natural food production per year per hm^2^ of nationally averaged production of farmland, the significance of which is to reflect the potential capacity of the ecosystem to contribute to the relative magnitude of ecological services. In this regard, the economic value of natural food production per year is determined based on the food production per hm^2^ and food prices in that year. Due to time differences, it is necessary to adjust the equivalent factors, i.e., the individual values in Table 1, according to the NPP per unit area, precipitation and soil conservation in that year. The adjustment method is as follows:

$$F_{nij}=\left\{\begin{array}{c} P_{ij}\times F_{n1}\\ R_{ij}\times F_{n2}\\ S_{ij}\times F_{n3} \end{array}\right.$$

where *F_nij_* is the unit area value equivalent factor of the *n*th ecological service function of an ecosystem in month *j* of region *i*; *P_ij_* is the NPP spatial and temporal regulator in month *j* of region *i* of the ecosystem; *R_ij_* is the precipitation spatial and temporal regulator in month *j* of area *i* of the ecosystem; *S_ij_* is the soil conservation spatial and temporal regulator in month *j* of region *i* of the ecosystem; *F_n_*_1_ is the national average annual value per unit area equivalent factor for the food production, raw material production, gas regulation, climate regulation, environmental purification, nutrient cycling, biodiversity maintenance, or aesthetic landscape service of this type of the ecosystem; *F_n_*_2_ is the national average annual value per unit area equivalent factor for the water supply or hydrological regulation service function of this type of ecosystem; and *F_n_*_3_ is the national average annual value per unit area equivalent factor for the soil conservation service function of this type of ecosystem.

$$P_{ij}=B_{ij}/\bar{B}$$

where *B_ij_* is the NPP in month *j* of area *i* of the ecosystem (t·km^−2^), $\bar{B}$ is the national average annual NPP of the ecosystem (t·km^−2^).

$$R_{ij}=W_{ij}/\bar{W}$$

where *W_ij_* is the average precipitation per unit area in month *j* of region *i* (mm·km^−2^), $\bar{W}$ is the national average annual precipitation per unit area (mm·km^−2^).

$$S_{ij}=E_{ij}/\bar{E}$$

where *E_ij_* is the soil conservation simulation in month *j* of region *i* of the ecosystem (t·km^−2^), $\bar{E}$ is the national average soil conservation simulation per unit area (t·km^−2^).

The ESV was calculated using the equation as follows:

$$\mathrm{ESV}=\sum (A_{n}\times F_{nij})$$

where *A_n_* represents the total area (km^2^) of the *n*th ecological service function of an ecosystem.

The spatial distribution of the total ESV in the study area at grid scale with a resolution of 1 km × 1 km was calculated, and the township, county, regional boundary and land-use data were overlaid with the corresponding total ESV, and the zonal tool in Arc GIS spatial analyst tools was used for the chunking statistics to obtain the total ESV at four other spatial scales in 2015 and 2020 in the mountainous areas of the BTH region. Other processing and analyses of the data was conducted on Microsoft Excel 2016.

## 3. Results 

### 3.1. Total ESV at Different Spatial Scales

The total ESV in 2015 and 2020 in the mountainous areas of BTH region is 5671 × 10^8^ CNY and 6381 × 10^8^ CNY, respectively. The total ESV increased by 12.5% from 2015 to 2020.

#### 3.1.1. Total ESV at Grid Scale

**Figure 2:**
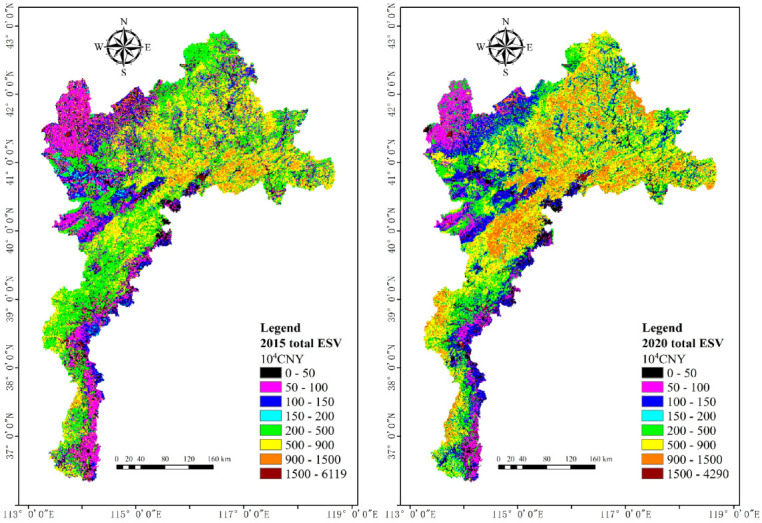
Spatial distribution of the total ESV at grid scale in 2015 and 2020.

For grid scale, we divided the range of total ESV in 2015 and 2020 into eight intervals, including 0–50, 50–100, 100–150, 150–200, 200–500, 500–900, 900–1500 and >1500 × 10^4^ CNY (Figure 2). Although the maximum value of total ESV is 6119 × 10^4^ CNY in 2015, which is larger than that in 2020 (4290 × 10^4^ CNY), the mean value of total ESV is 465.5 × 10^4^ CNY in 2015, which is smaller than that in 2020 (523.6 × 10^4^ CNY). Specifically, the overall spatial distribution of the total ESV in both 2015 and 2020 showed a lower value in the northwest and mountain-plain junction in the southeast of the study area, mainly distributed between 50–150 × 10^4^ CNY, and a higher value in the northeast, mainly distributed between 500–1500 × 10^4^ CNY. Additionally, from 2015 to 2020, the area occupied by the interval 100–150 × 10^4^ CNY in the northwest and mountain-plain junction in southeast and the area occupied by the interval 900–1500 × 10^4^ CNY in the northeast increased significantly, which was initially inferred to be the main reason for the increase of the total ESV in 2020 compared with 2015.

**Figure 3:**
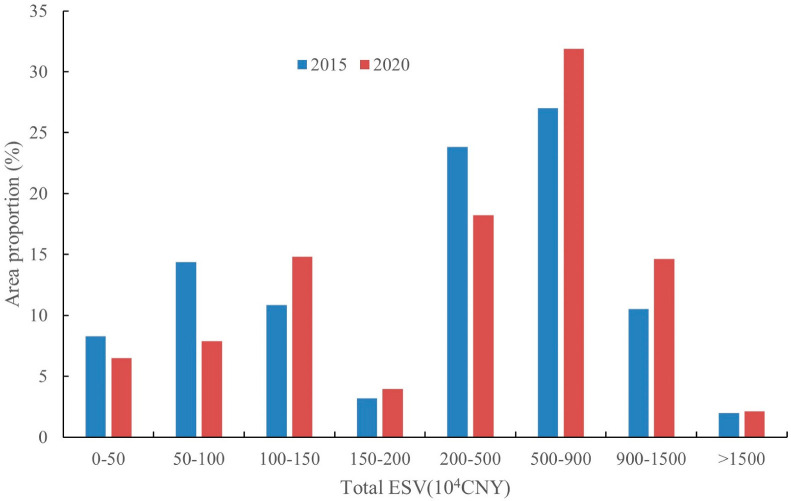
Area proportion total ESV of study area at grid scale in 2015 and 2020.

Further, we calculated the area proportion of the study area occupied by each interval of total ESV (Figure 3). Although the distribution area of the total ESV below 100 × 10^4^ CNY decreased from 2015 to 2020, the distribution area in the high value range (500–1500 × 10^4^ CNY) of total ESV showed the same upward trend, and the growth rates were all 15% and above. It can be concluded that, at the grid scale, there is a trend of conversion from low to high values of total ESV from 2015 to 2020.

#### 3.1.2. Total ESV at Township Scale

**Figure 4:**
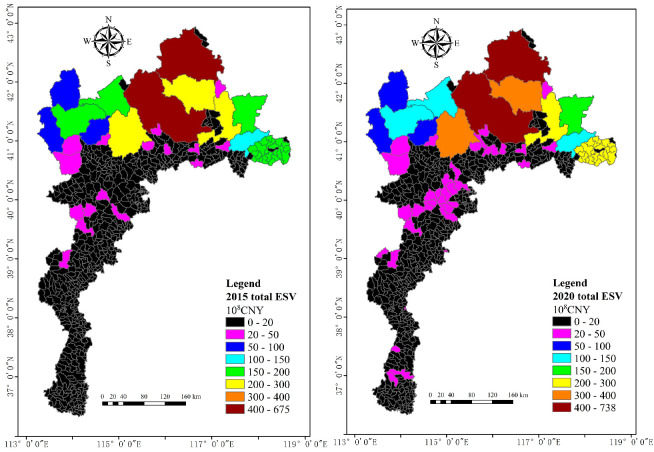
Spatial distribution of the total ESV at township scale in 2015 and 2020.

The total ESV was calculated for 548 townships in the study area in 2015 and 2020, and we divided the range of total ESV into eight intervals, including <20, 20–50, 50–100, 100–150, 150–200, 200–300, 300–400 and >400 × 10^8^ CNY (Figure 4). For township scale, the total ESV are 0 and 675 × 10^8^ CNY for the minimum and maximum values in 2015 and 0 and 738 × 10^8^ CNY in 2020, respectively.

The spatial distribution of total ESV showed that the low values were distributed in about 425 townships in the center and south, mainly at 20 × 10^8^ CNY and below, and—with the exception of about 10 townships in the center, which grew from 0–20 to 20–50 × 10^8^ CNY—the remaining townships remained largely unchanged from 2015 to 2020. Meanwhile, about six townships in the northeast show high values, mainly at 150 × 10^8^ CNY and above in 2015 and 2020, and the total ESV in some townships of the northeast increased from 150–200 to 200–300 × 10^8^ CNY.

**Figure 5:**
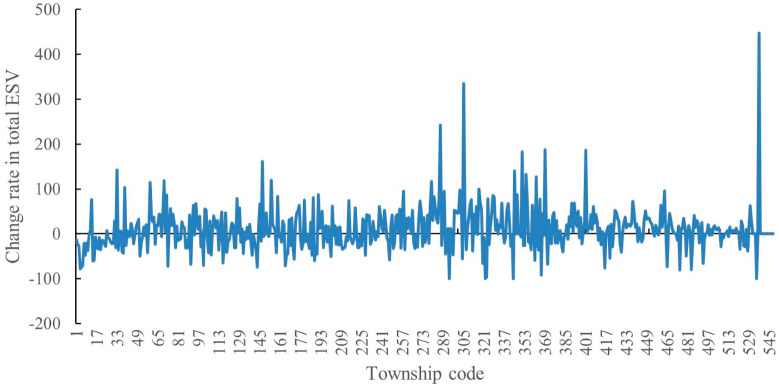
Change rate of total ESV of study area at township scale from 2015 to 2020.

We calculated the change rate of the total ESV in each township from 2015 to 2020 (Figure 5). As shown in the figure, the total ESV shows an increasing and decreasing trend in 309 and 225 counties, respectively, and the total ESV in about 14 counties remained unchanged from 2015 to 2020. The main change rate is 35%. Although the growth rate for the whole study area is 12.5%, the total ESV at the township scale varies drastically.

#### 3.1.3. Total ESV at County Scale

**Figure 6:**
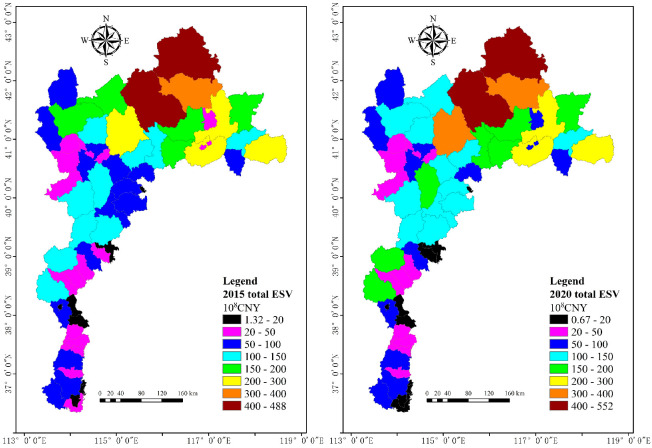
Total ESV of study area at county scale in 2015 and 2020.

The study area includes a total of 94 counties, some of which have only small areas located within the study area; removing these gives us 58 counties whose jurisdictional boundaries are mainly or entirely within the study area. We obtained the spatial distribution of total ESV with zonal statistics for these 58 counties, and we divided the range of total ESV into eight intervals shown in Figure 4 (Figure 6). For county scale, the total ESVs are 1.32 and 488 × 10^8^ CNY for the minimum and maximum values in 2015 and 0.67 and 552 × 10^8^ CNY in 2020, respectively. The maximum values are lower than those at the township scale.

The spatial distribution of total ESV show that the low values are distributed in about five counties in the northwest and 15 counties in the south, mainly at 100 × 10^8^ CNY and below. While about nine counties in the northeast show high values, mainly at 150 × 10^8^ CNY and above in 2015 and 2020, which is the same as township scale. The total ESV in about seven counties in the center increased from 50–150 to 100–200 × 10^8^ CNY from 2015 to 2020.

**Figure 7:**
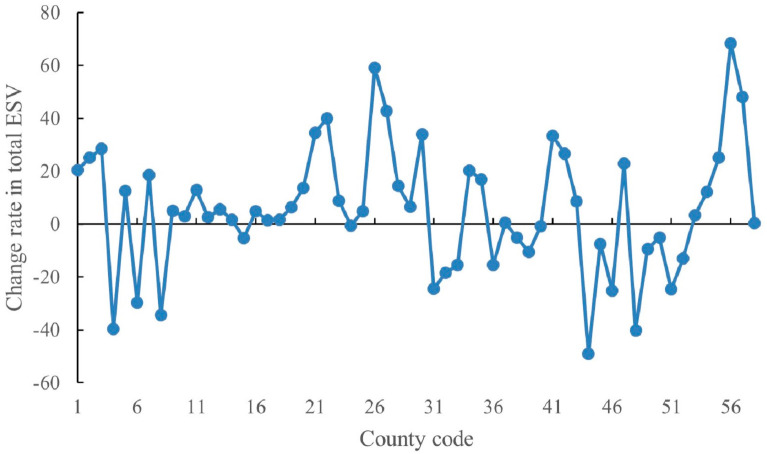
Change rate of total ESV of study area at county scale from 2015 to 2020.

Similarly, the change rate of the total ESV in each county from 2015 to 2020 was also obtained (Figure 7). From 2015 to 2020, the total ESV shows an increasing and decreasing trend in 38 and 20 counties, respectively, with a rate of change of around 20%. It can be seen that the change is more pronounced at the county scale, while the degree of change in total ESV at the county scale is lower than that at the township scale.

#### 3.1.4. Total ESV at Region Scale

**Figure 8:**
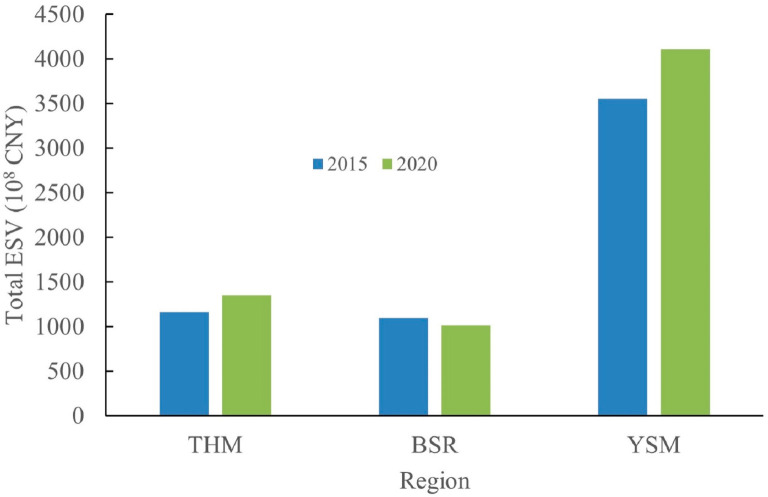
Total ESV of study area at region scale in 2015 and 2020 (THM—Taihang Mountain, BSR—Bashang region, YSM—Yanshan Mountain).

As mentioned previously, the mountainous areas in BTH region were divided into three regions, namely THM, BSR and YSM. As shown in Figure 8, among the three regions, the total ESV is largest in YSM, which is 3552 × 10^8^ CNY in 2015, accounting for 61.11% of the total value and grows to 4111 × 10^8^ CNY in 2020, accounting for 63.46% of the total value. The total ESV of THM and BSR is not much different, both around 1100 × 10^8^ CNY, accounting for 19.98% and 18.90% of the total value in THM and BSR in 2015, respectively, and accounting for 20.86% and 15.67% of the total value in THM and BSR in 2020, respectively. The total ESV in both THM and YSM increases by 16% from 2015 to 2020, while that in BSR decreases by about 8%. In terms of total ESV per unit area, this is 1.5–2 times higher in YSM than THM and BSR, and it is inferred that people receive more life support products and services directly or indirectly through ecosystem structures, processes and functions in YSM than in THM and BSR.

#### 3.1.5. Total ESV at Ecosystem Scale

According to the calculation results, the main types of ecosystems in the mountainous areas of BTH region are farmland and woodland, both of which account for about 30% of the entire region in 2015 and 2020 (Table 2). For the total ESV, in 2015, the total ESV of woodland is highest, at 2513 × 10^8^ CNY, accounting for 43.17% of the total value, followed by grassland and farmland, accounting for 29.75% and 19.08% of the total value, respectively. Water body and bare land are less, accounting for 4.85% and 1.93% of the total value, respectively, and construction land provides the lowest value of services, accounting for only 1.21% of the total value (Table 2).

Despite the fact that the woodland area grows by only 1.51% in the mountainous areas of the BTH region in 2020, the total ESV grows by 28.65%, accounting for 50.14% of the total value. The area of farmland decreases by 7.25%, but the total ESV remains essentially unchanged. In contrast, the area of grassland, water body and bare land all show different degrees of decrease, with rates of 3.54%, 3.71% and 26.50%, respectively. Correspondingly, the total ESV also show different degrees of decrease, with rates of 2.31%, 32.86% and 58.04%, respectively. At the same time, the area of construction land shows a significant increase, and accordingly, the total ESV also shows growth at a rate of 145.71%. Although the total ESV of each ecosystem changes to different degrees, for the mountainous areas of the BTH region, woodland still has the highest total ESV in 2020, with a value of 3233 × 10^8^ CNY, accounting for 50.14% of the total value, followed by grassland and farmland, accounting for 26.24% and 17.27% of the total value, respectively, while water body decreases to the same level as construction land, both accounting for 2.67% of the total value. The smallest value is bare land, accounting for only 0.72% of the total value (Table 2).

### 3.2. Values of Ecosystem Service Categories

**Figure 9:**
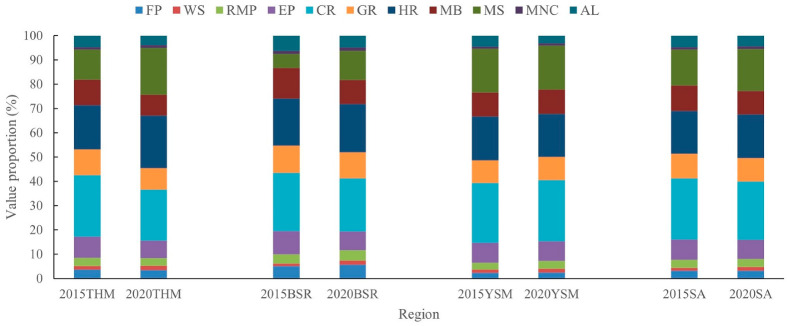
The value proportion of ecosystem service categories in the study area, THM, BSR and TSM in 2015 and 2020 (THM—Taihang Mountain, BSR—Bashang region, YSM—Yanshan Mountain, SA—study area; FP—food production, WS—water supply, RMP—raw material production, EP—environmental purification, CR—climate regulation, GR—gas regulation, HR—hydrological, MB—maintain biodiversity, MS—maintain soil, MNC—maintain nutrient cycle, AL—aesthetic landscape).

Ecosystem service categories included supply services, namely food production, raw material production and water supply services; regulatory services, namely gas regulation, climate regulation, environmental purification and hydrological regulation services; support services, namely soil maintenance, nutrient cycle maintenance and biodiversity maintenance services; and cultural services, namely aesthetic landscape. In terms of ecosystem service categories, for 2015 in the study area, the regulatory services have the highest value, with the climate regulation service ranking first, at 1411 × 10^8^ CNY, and hydrological regulation ranking second, at 989 × 10^8^ CNY, with the value of those two services accounting for 25.19% and 17.65% of the total value, respectively. Soil maintenance service was third, accounting for 14.78% of the total service value, followed by biodiversity maintenance, gas regulation and environmental purification services, which together account for 28.92% of the total service value, the value of all other ecological services was lower, accounting together for 13.46% of the total service value (Figure 9).

For 2020 in the study area, the ranking of all ecosystem services categories as a percentage of the total value remains the same, with only some changes in the accounting percentages. Climate regulation service is still in first place, accounting for 24.02% of the total value, followed by hydrological regulation, accounting for 17.93% of the total value. However, it is worth mentioning that, except for the value of cultural services, the value of all ecosystem service categories has increased to varying degrees relative to 2015. Among these, the value of the water supply service increased the most, with a growth rate of 46.35%, followed by soil service maintenance, with a growth rate of 32.27%.

For THM, in 2015, the climate regulation service has the highest value with 25.39% of the total value, followed by the hydrological regulation service with 18.22%. The lowest are the water supply and nutrient cycle maintenance services with 1.36% and 1.14%, respectively. In 2020, the hydrological regulation service has the highest value with 21.61% of the total value, the climate regulation service is second with 21.05% of the total value, and the lowest are still water supply and nutrient cycle maintenance services, with 1.82% and 1.08%, respectively. Unlike the study area, the value of supply services in THM all increased, among which the value of the water supply service had a large increase with a growth rate of 55.29%. The value of the soil maintenance service increased the most, with a growth rate of 81.57%, and the value of the hydrological regulation service also increased somewhat, with a growth rate of 37.99%. The values of the other three regulation services, biodiversity maintenance and cultural services showed a small decrease, at a rate of 3%.

For BSR, both in 2015 and 2020, the climate regulation and hydrological regulation services are ranked first and second in value, accounting for 24.05% and 19.35% of the total value in 2015 and 21.97% and 19.82% in 2020, respectively. The lowest value of the water supply service was only 1.09% of the total value in 2015 and the lowest value of the nutrient cycle maintenance service was 1.43% of the total value in 2020. Compared with 2015, the value of supply services on the BSR in 2020 all increased, including the value of the water supply service, which increased by 48.64%. The value of regulating services all decreased, including the value of the environmental purification service, which decreased by 24.63%. The value of the soil maintenance service continued to increase the most among all services, with an increase rate of 93.63%, however, the value of the biodiversity maintenance and cultural services both decreased at a rate of about 28%.

For YSM, both in 2015 and 2020, climate regulation and hydrological regulation services are dominant, and the value of water supply and nutrient cycle maintenance services are the lowest, with values are similar in proportion to the total values of THM and BSR. Except for cultural services, the value of the other ecosystem services increased from 2015 to 2020, with the supply of all services and nutrient cycle maintenance service dominating, with growth rates of 23% and above.

In general, the proportion of each ecosystem service value and the main ecosystem service functions are basically the same for the mountainous areas of BTH, the THM, the BSR and the YSM. In both 2015 and 2020, climate regulation and hydrological regulation services were dominant, and water supply and nutrient cycle maintenance services had the lowest values.

However, the change trend of each ecosystem service value from 2015 to 2020 was dominated by increasing in the mountainous area of BTH and the YSM, among which, the value of the water supply service, soil maintenance service and nutrient cycle maintenance service in the mountainous area of BTH had growth rates of 46.35%, 32.27% and 19.17%, respectively. The YSM are dominated by the growth of the water supply service, nutrient cycle maintenance, raw material production, and food production services, with growth rates of 29.86%, 28.54%, 24.03% and 23.01%, respectively. The change pattern of the value of ecosystem service categories in THM and BSR is basically the same, with the value of the soil maintenance and water supply services increasing the most, with growth rates of 49% and above, and the value of the regulating services basically showing a decreasing trend.

### 3.3. Values of Ecosystem Service Categories for Different Ecosystems

We further obtained value proportions of 11 ecosystem service categories corresponding to each ecosystem (Table 3), the unit in Table 3 is %.

The proportion of the total value of regulatory services for all ecosystems was the highest in both 2015 and 2020, with the regulatory services of woodland being the largest among all ecosystems, at 24.89% in 2015 and 28.51% in 2020. In contrast, the lowest value proportion of regulating services was accounted for by construction land and bare land, with a value proportion of 0.83% in 2015 for construction land and of 0.55% in 2020 for bare land. Cultural services have the lowest value proportion among those four primary ES types, with the value proportions of cultural services for water body, construction land and bare land in particular less than 0.3%. Among the regulatory services, the largest proportions for climate regulation were in farmland, woodland and grassland, and the largest proportions for hydrological regulation were in water body, construction land and bare land. In terms of the 11 ecosystem service categories, with the exception of the food production service, which has the highest value proportion in farmland, all other ecosystem services are highest in woodland. The value proportion of the nutrient cycle maintenance service was less than 0.5% among the six LULC types in 2015 and 2020, which is the lowest. In summary, it can be seen that from 2015 to 2020, in the various ecosystems of mountainous areas of the BTH region, the main ecosystem services are regulatory services. Furthermore, the main regulatory services of farmland, woodland and grassland are climate regulation services. Meanwhile, the main regulatory services for water body, construction land and bare land are hydrological regulation services. This is followed by support services, which are mainly represented by the soil maintenance service for woodland and grassland, and the biodiversity maintenance service for the other ecosystems, while the nutrient cycle maintenance service is lowest.

## 4. Discussion

### 4.1. Variability of the Total ESV at Different Spatial Scales

**Figure 10:**
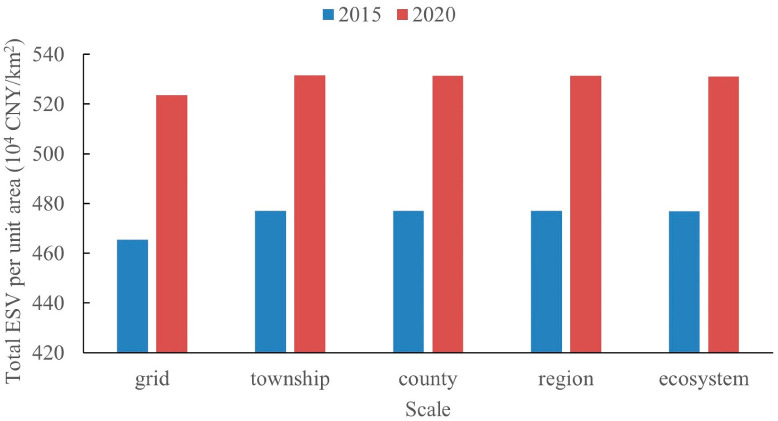
The total ESV per unit area of study at different scales in 2015 and 2020.

Scale refers to the range or frequency of geographical phenomena or ecological processes under certain spatial and temporal conditions. It is important in geography and ecology research [80]. Generally, the administrative boundaries of townships and counties may disrupt the natural environment’s largely complete and similar distribution, resulting in a failure to adequately reflect biophysical processes [81]. Conversely, a region or an ecosystem is a relatively complete system with similar natural and social attributes [82], which is the most important ecological process boundary [83]. Moreover, we also considered the pixel scale, that is, a 1 km × 1 km grid scale. Figure 10 presents the changes of the total ESV per unit area in 2015 and 2020 at different spatial scales, and it can be seen from the figure that the total ESV per unit area in 2015 and 2020 calculated at the township, county, region and ecosystem scales are basically equal and larger than the results obtained at the grid scale.

This is because the calculation of the total ESV per unit area of the study area at the grid scale is obtained by averaging the total ESV per unit area of all grids, i.e., 121,819 grids, while the township, county, regional, and ecosystem scales are calculated by averaging the total ESV per unit area of each township, county, region, and ecosystem separately, multiplying by the corresponding area, summing, and dividing by the total area corresponding to each scale. Statistically, it is usually considered that using the average value does not always truly reflect the actual variability of the data, especially when the sample variance is relatively large. From this, we may infer that the reason why the total ESV per unit area calculated at the grid scale is lower than the remaining four scales is due mainly to the large spatial variation of the total ESV in the study area. At the same time, the results of the other four scales are not very different, especially when divided into three regions, indicating that the total ESV varies greatly among the THM, BSR and YSM regions, which we also confirmed in Section 3.1.4. This leads to the conclusion that the calculation of ESV can be rendered more accurate by considering climate and vegetation conditions by region, ecosystem, county or township.

**Figure 11:**
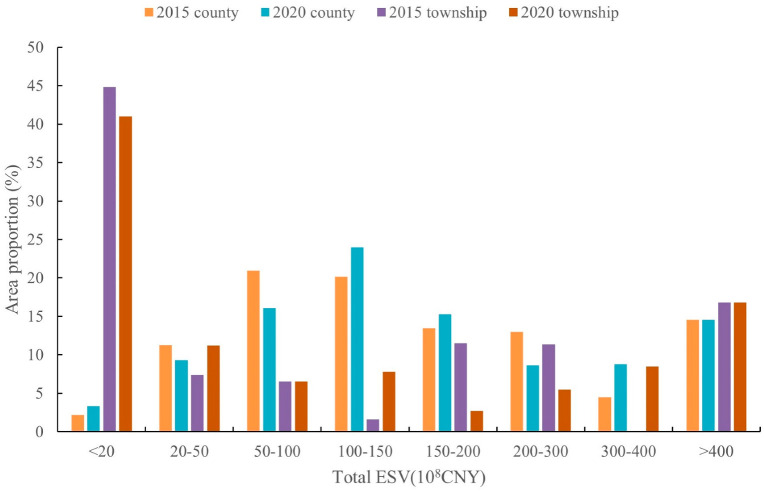
Area proportion ESV totals of the study area at township and county scales in 2015 and 2020.

Further, scientific information related to the changes in the ESV across different scales is vital for land use and ecosystem management [84]. Both administrative and natural boundaries were used to simulate the scale effect characteristics of ESV, rather than using single scale, allowing us to provide more accurate information on ecosystem service management for decisionmakers at different scales across the mountainous area of BTH. The results show that the same policies implemented at one spatial scale do not necessarily produce similar results at other scales [85]. Although the total ESVs calculated at the township and county scales in 2015 and 2020 are basically equal, they are 5801 × 10^8^ CNY at the township scale and 5795 × 10^8^ CNY at the county scale in 2015, and 6464 × 10^8^ CNY at the township scale and 6454 × 10^8^ CNY at the county scale in 2020, respectively. Thus, there is a difference of only 0.1–0.15%, indicating that the ESV totals at those spatial scales differ significantly in distribution area proportion for the same total ESV interval segment. The township scale is mainly dominated by ESVs of less than 20 × 10^8^ CNY, which accounts for about 40% of the whole study area, followed by ESVs of greater than 400 × 10^8^ CNY at 15% (Figure 11). Meanwhile, the distribution area proportion of these two intervals is basically unchanged from 2015 to 2020. The changes were mainly concentrated between 100–400 × 10^8^ CNY, and from 2015 to 2020 showed change characteristics indicating that the ESV totals that were located in the intervals 100–150 and 300–400 × 10^8^ CNY increased and those in the 150–300 × 10^8^ CNY interval decreased, with change rates of basically 10%. For the county scale, the total ESV distribution intervals are mainly 50–150 × 10^8^ CNY, and the area proportion of the whole study area is also about 40%, with the least area proportion being that for the total ESV distribution in the interval of less than 20 × 10^8^ CNY. In addition, unlike the township scale, the change rate of the area proportion occupied by each interval of the total ESV distribution from 2015 to 2020 is less than 5%. It is thus presumed that, for calculating the interannual variation of total ESV, the spatial scale variability is large. The township scale may be the ideal unit of ecosystem management as it can reflect the spatial and temporal variation of ESV totals more realistically, which is consistent with the results of Liu et al. [84]. Despite the fact that the variation of ESV totals is more noticeable at the grid scale of 1 km × 1 km than at the township grid, the number of grids is too great for management.

### 4.2. Interaction between LULC and ESV

There was a mutual interaction relationship between LULC and ESVs [24]. LULCC is the major driving force for the spatial pattern of the ESV through its influence on the ecosystem structure and function that change the ESV per unit area [25]. In contrast, the transformation of ecosystem services shows a complex impact on the mode and efficiency of land use. The ESV per unit area is higher in areas with vegetation growth and lower in the opposite direction [26]. Our results demonstrate that woodland is the most likely to cause ESV changes among all land use types, with a 1.51% increase in woodland area bringing 28.65% increase in the ESV of woodland, followed by water body, with a 3.71% decrease in water body area also causing 32.86% decrease in ESV of water body, while a 7.25% decrease in farmland area leaves its ESV essentially unchanged. Land use degree was negatively correlated with ESV [86], with higher land use degrees associated with reduced ecosystem integrity and functioning. Therefore, farmland, with high land use degree, had low ESV, while woodland and water body, with low land use degree, had high ESV [45]. It is inferred that for the mountainous areas of the BTH region, the ecosystem service of woodland and water body are more prominent compared with other land use types.

To further confirm this, the value of the 11 ecosystem service categories was analyzed. Woodland showed a trend of increasing change from 2015 to 2020, where the value of the food production service increasing by 55.84% from 38.80 × 10^8^ CNY in 2015 to 60.46 × 10^8^ CNY. This is in exception of hydrological regulation, which increased at 7.57%, and the rest of the services, which increased at between 15.43% and 45.61%. Farmland showed a decrease in the value of ecosystem services, which were all 15% or below. Water body showed a decrease in the value of all ten ecosystem service categories, except for hydrological regulation. This is particularly true of aesthetic landscape and environmental purification services, which decreased by more than 60%, and all other services, which decreased by 13.89–58.64%.

**Figure 12:**
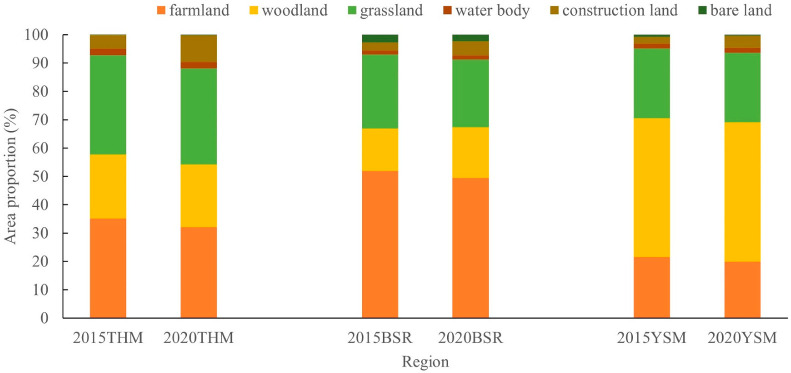
Area proportion of LULC in THM, BSR and YSM in 2015 and 2020 (THM—Taihang Mountain, BSR—Bashang region, YSM—Yanshan Mountain).

Similarly, the main LULC type in YSM is woodland, with a distribution area of 49% both in 2015 and 2020, while the main LULC types in THM and BSR are farmland and grassland. The distribution area of woodland is between 15% and 20%, with a certain similarity in LULC distribution, and a greater difference from YSM (Figure 12). According to 3.2, the value change trend of each ecosystem service category in YSM and THM differs greatly, while the change patterns of the value of the ecosystem service categories in THM and BSR are basically the same. There is some similarity between the value changes of ecosystem service categories and the distribution of LULC types.

### 4.3. Limitations and Further Study

In this study, the ESV estimation model based on the equivalence factor method can express ESV in monetary terms and is easy to operate, but the model also has some limitations [87] such as ignoring the differences in results of the same LULC type due to the different environments in which it is located. Secondly, the temporal effect of ESV cannot be ignored, as studies have shown that the interannual variation of human activities such as reforestation in a climatic context has a different impact on ES changes than the spatial variation of human activities [88]. Therefore, the study of the relationship between human activities and ESV needs to be deepened [89]. In addition, after developing an understanding of the spatial distribution of ESV, the next step is to conduct relevant research on regional payments for ES, which is an important policy tool for coordinating ecological protection and regional socioeconomic development [90]. This will help better provide guidance for coordinated regional socio-ecological development and land use management.

Meanwhile, there are questions about the relationship between total ESV and human wellbeing that do not involve scale. Whether it is grid, township, county or regional, there are only spatial differences in the high or low ESVs, which may affect the high or low benefits to people. If this aspect of the discussion is to take place, we believe that it should involve the different needs of people for ecosystems in different regions. As in the MA concept, human wellbeing refers to the basic material conditions required to maintain a high quality of life, health, safety and security, good social relationships, and freedom of choice and action [10]. Wellbeing enhancement is related to the ability to meet people’s basic needs and capabilities, the resources and opportunities they have access to, and their physical, social, and spiritual conditions [91]. These different needs for ecosystems, in turn, influence the ESV. Ecosystems therefore provide goods and services for human wellbeing, and in turn, preferences for human wellbeing can feed back directly or indirectly into ecosystem management, thereby changing or influencing the supply of primary ES [92]. This then also implies that we need to further investigate the impact of specific categories of ES, including provisioning, regulating, supporting, and cultural services, on multiple layers of human wellbeing [10,93], i.e., objective wellbeing—such as physical—economic and social, and subjective wellbeing.

## 5. Conclusions

Ecosystem services, which are closely related to human wellbeing, have a high potential value. Although it is difficult to identify, quantify and monetize the value of ecosystem services, adequate valuation of ecosystem service values is now an urgent need for the implementation of policy measures such as ecosystem asset management, ecological compensation and paid use of ecosystem services. The total ESV in 2015 and 2020 in the mountainous areas of BTH region is 5671 × 10^8^ CNY and 6381 × 10^8^ CNY, respectively. Both in 2015 and 2020, climate regulation and hydrological regulation services were dominant, and water supply and nutrient cycle maintenance services had the lowest values in the mountainous area of BTH, the THM, the BSR and the YSM.

Meanwhile, ESs exhibit complex interconnections and strong scale characteristics. The calculation of ESVs can be made more accurate by considering climate and vegetation conditions at the regional, ecosystem, county or township scales rather than at grid scale, and for calculating the interannual variation of total ESV, the spatial scale variability is large. The assessment of ESVs is important from the point of view of planning the use of the environment, spatial planning, and nature conservation. On the other hand, the regional variability of ESV shows that people receive more life support products and services directly or indirectly through ecosystem structures, processes and functions in YSM than in THM and BSR.

The ecosystem services of woodland and water body are more prominent compared with other land use types, while construction land and bare land provide the lowest value of service. There is some similarity between the value changes of ecosystem service categories and the distribution of LULC types, indicating that ESV can directly reflect the vegetation coverage in a region. Therefore, land use managers may more effectively control resources with knowledge of the spatial distribution of ESV.

## Figures and Tables

**Table 1:** The equivalents of ecosystem service value supplied by per unit area of ecosystem in China (Xie et al. [68]).

Ecosystem Types		Supply Service			Regulatory Service				Support Service			Cultural Service
Primary category	Secondary category	FP *	RMP	WS	GR	CR	EP	HR	MS	MNC	MB	AL
Farmland	Dryland	0.85	0.40	0.02	0.67	0.36	0.10	0.27	1.03	0.12	0.13	0.06
	Paddy field	1.36	0.09	−2.63	1.11	0.57	0.17	2.72	0.01	0.19	0.21	0.09
Woodland	Coniferous forest	0.22	0.52	0.27	1.70	5.07	1.49	3.34	2.06	0.16	1.88	0.82
	Mixed coniferous forest	0.31	0.71	0.37	2.35	7.03	1.99	3.51	2.86	0.22	2.60	1.14
	Broadleaf forest	0.29	0.66	0.34	2.17	6.50	1.93	4.74	2.65	0.20	2.41	1.06
	Shrubland	0.19	0.43	0.22	1.41	4.23	1.28	3.35	1.72	0.13	1.57	0.69
Grassland	Steppe	0.10	0.14	0.08	0.51	1.34	0.44	0.98	0.62	0.05	0.56	0.25
	Scrub	0.38	0.56	0.31	1.97	5.21	1.72	3.82	2.40	0.18	2.18	0.96
	Meadow	0.22	0.33	0.18	1.14	3.02	1.00	2.21	1.39	0.11	1.27	0.56
Wetland	Wetland	0.51	0.50	2.59	1.90	3.60	3.60	24.23	2.31	0.18	7.87	4.73
Bare land	Desert	0.01	0.03	0.02	0.11	0.10	0.31	0.21	0.13	0.01	0.12	0.05
	Bare land	0.00	0.00	0.00	0.02	0.00	0.10	0.03	0.02	0.00	0.02	0.01
Water body	Water	0.80	0.23	8.29	0.77	2.29	5.55	102.24	0.93	0.07	2.55	1.89
	Glacial snow	0.00	0.00	2.16	0.18	0.54	0.16	7.13	0.00	0.00	0.01	0.09

* FP—food production, WS—water supply, RMP—raw material production, EP—environmental purification, CR—climate regulation, GR—gas regulation, HR—hydrological, MB—maintain biodiversity, MS—maintain soil, MNC—maintain nutrient cycle, AL—aesthetic landscape.

**Table 2:** Total ESV of study area at ecosystem scale in 2015 and 2020.

Ecosystem Type		Farmland	Woodland	Grassland	Water Body	Construction Land	Bare Land
Area/km^2^	2015	39,332	41,824	33,540	2319	3689	1313
	2020	36,481	42,454	32,353	2233	6921	965
Area proportion/%	2015	32.40	34.44	27.63	1.91	3.04	1.08
	2020	30.05	34.97	26.65	1.84	5.70	0.79
Total ESV/10^8^ CNY	2015	1111	2513	1732	283	70	112
	2020	1114	3233	1692	190	172	47
Value proportion/%	2015	19.08	43.17	29.75	4.85	1.21	1.93
	2020	17.27	50.14	26.24	2.67	2.67	0.72

**Table 3:** The value proportions of 11 specific ESVs of different LULC in 2015 and 2020.

Primary ES Type	Secondary ES Type	Farmland		Woodland		Grassland		Water Body		Construction Land		Bare Land		Total	
		15 *	20 *	15	20	15	20	15	20	15	20	15	20	15	20
Supply service	Food production	1.43	1.19	0.67	0.94	0.81	0.80	0.09	0.05	0.08	0.16	0.05	0.03	3.13	3.17
	Raw material production	0.93	0.82	1.15	1.48	0.99	0.86	0.06	0.04	0.05	0.11	0.04	0.02	3.22	3.32
	Water supply	0.11	0.34	0.55	0.57	0.32	0.40	0.22	0.17	0.02	0.07	0.05	0.03	1.28	1.59
Regulatory service	Gas regulation	2.40	1.94	3.74	4.60	3.29	2.52	0.21	0.10	0.13	0.27	0.13	0.05	9.89	9.48
	Climate regulation	4.52	3.66	10.64	12.93	8.45	6.24	0.52	0.23	0.24	0.53	0.25	0.09	24.62	23.67
	Environmental purification	1.68	1.33	3.3	3.98	2.85	2.12	0.37	0.11	0.11	0.19	0.21	0.06	8.53	7.79
	Hydrological regulation	3.31	3.98	7.21	7.00	4.13	4.5	2.84	2.13	0.35	0.78	0.54	0.36	18.39	18.75
Support service	Soil maintenance	1.29	1.31	9.65	11.01	3.46	4.51	0.07	0.08	0.04	0.21	0.02	0.01	14.52	17.13
	Nutrient cycle maintenance	0.30	0.27	0.36	0.47	0.33	0.31	0.02	0.01	0.02	0.04	0.01	0.01	1.04	1.11
	Biodiversity maintenance	2.10	1.61	4.09	4.96	3.54	2.60	0.29	0.11	0.12	0.23	0.40	0.10	10.53	9.60
Cultural service	Aesthetic landscape	1.00	0.77	1.81	2.20	1.59	2.12	0.16	0.11	0.06	0.19	0.23	0.06	4.85	4.39

* 15 represents 2015 and 20 represents 2020.

## Data Availability

All data, models, and code generated or used during the study appear in the submitted article.
